# Neonatal malaria in Nigeria -a 2 year review

**DOI:** 10.1186/1471-2431-6-19

**Published:** 2006-06-12

**Authors:** Iyabo T Runsewe-Abiodun, Olusoga B Ogunfowora, Bolanle M Fetuga

**Affiliations:** 1 Department of Paediatrics, Olabisi Onabanjo University Teaching Hospital, PMB 2001, Sagamu, Ogun State, South-west, Nigeria

## Abstract

**Background:**

In view of the fact that a significant proportion of neonates with malaria may be missed on our wards on the assumption that the disease condition is rare, this study aims at documenting the prevalence of malaria in neonates admitted into our neonatal ward. Specifically, we hope to describe its clinical features and outcome of this illness. Knowledge of these may ensure early diagnosis and institution of prompt management.

**Methods:**

Methods Hospital records of all patients (two hundred and thirty) admitted into the Neonatal ward of Olabisi Onabanjo University Teaching Hospital, Sagamu between 1st January 1998 and 31^st ^December 1999 were reviewed. All neonates (fifty-seven) who had a positive blood smear for the malaria parasite were included in the study. Socio-demographic data as well as clinical correlates of each of the patients were reviewed. The Epi-Info 6 statistical software was used for data entry, validation and analysis. A frequency distribution was generated for categorical variables. To test for an association between categorical variables, the chi-square test was used. The level of significance was put at values less than 5%.

**Results:**

Prevalence of neonatal malaria in this study was 24.8% and 17.4% for congenital malaria.

While the mean duration of illness was 3.60 days, it varied from 5.14 days in those that died and and 3.55 in those that survived respectively. The duration of illness significantly affected the outcome (p value = 0.03). Fever alone was the clinical presentation in 44 (77.4%) of the patients. Maturity of the baby, sex and age did not significantly affect infestation. However, history of malaria/febrile illness within the 2 weeks preceding the delivery was present in 61.2% of the mothers. Maternal age, concurrent infection and duration of illness all significantly affected the outcome of illness. Forty-two (73.7%) of the babies were discharged home in satisfactory condition.

**Conclusion:**

It was concluded that taking a blood smear to check for the presence of the malaria parasite should be included as part of routine workup for all neonates with fever or those whose mothers have history of fever two weeks prior to delivery. In addition, health education of pregnant mothers in the antenatal clinic should include early care-seeking for newborns.

## Background

Malaria has been recognised as a leading cause of infant morbidity and mortality in Africans [1] but neonatal malaria was thought to be rare. This was thought to be partly due to the protective effect of Hb F on the neonates [2]. Recent report however seems to suggest that malaria is not as rare among newborn infants in Sub-Saharan Africa as previously thought. In a series, prevalence of congenital malaria was put at 7% with a range of 0–23% [3]. Congenital malaria has been shown to occur in children of clinically healthy mothers who are delivered in malaria endemic- areas [4].

As clinical signs of neonatal malaria may be indistinguishable from that of neonatal sepsis, it has been suggested that screening for malaria parasite be included as part of routine investigation in newborn infants with fever [5]. Sometimes, the inability of clinicians to exercise that index of suspicion for congenital malaria has unwittingly increased the duration of hospital stay of the neonate or led to an increase in neonatal mortality.

The burden of malaria in non-malarious countries had led to the suggestion by workers that neonatal malaria is to be considered in those newborns with congenital infection born to mothers who had travelled to endemic areas, even when they appear clinically healthy [6,7].

A cursory observation on our neonatal ward suggests that malaria may not be uncommon in this environment. However, lack of scientific documentation has hindered the development of a definite policy on case management of neonatal malaria in our hospital.

Hence, this study aims at documenting the prevalence of diagnosed malaria in neonates admitted into our neonatal ward and description of its clinical features. In addition, we sought to determine the contributory factors to the outcome of the illness.

This we hope may ensure early diagnosis and institution of prompt management.

## Methods

This is a retrospective descriptive study. Case notes of all patients admitted into the Neonatal ward of Olabisi Onabanjo University Teaching Hospital, Sagamu, Ogun State, Western Nigeria between 1^st ^January 1998 and 31^st ^December 1999 were reviewed. This is the Teaching Hospital of the Olabisi Onabanjo University. The bed capacity of the neonatal ward is twenty-six (26).

Majority of the babies admitted into the ward are referred from the nearby general hospital and private hospitals within and outside the state. The only routine investigation in the unit is a sepsis work-up that is usually done in the service laboratory of the teaching Hospital. (Blood to be cultured are sent to the laboratory in 2 bottles, one containing the Glucose broth and the other the Thio-glycolate medium. This is incubated at 37°C for 48 hours. Growth in either of the medium is sub-cultured on Mc-Conkey, blood or chocolate agar and further tested for antibiotic sensitivity. However, if no growth is observed, further incubation is done for another 48 hours. Other investigations are determined by clinical presentations.

As with all other laboratory investigations in the hospital, blood smear for malaria parasite are examined in the service laboratory of the hospital by trained laboratory technologists; Thick and thin blood smear are examined after staining with Giemsa stain. Samples are considered negative after searching through 100 microscopic fields). All neonates whose record indicate positive blood smear for malaria parasite were included in this study. (no record of density of parasitemia was available).

Other data extracted from such baby's record included: age at onset of symptoms (days), sex, birth weight, length, place of delivery, presenting problem that immediately led to blood investigation for malaria parasite and duration of illness, assessed or known gestational age, as well as treatment regimen given and outcome of illness. Results of laboratory investigations viz: blood culture, full blood count and erythrocyte sedimentation rate were reviewed as well. The mothers' age, parity, history of febrile illness/malaria at least 2 weeks preceding the delivery and educational level were retrieved from the case notes.

For the purpose of the study, Neonatal malaria is defined as symptoms attributable only to malaria with evidence of ring forms of malaria parasite in the erythrocyte of an infant within the first twenty-eight days of life. Congenital malaria is defined as symptoms attributable only to malaria with evidence of ring forms of malaria parasite in the erythrocytes of an infant within the first seven days of life.

The Epi-Info 6 statistical software was used for data entry, validation and analysis. Frequency distribution was generated for categorical variables. To test for association between categorical variables, the chi-square test was used. Level of significance was put at values less than 5%.

## Results

Fifty-seven (24.8%) of the two hundred and thirty admissions into the neonatal ward in the period under review had a positive blood smear for malaria parasite. Eighty-nine out of the two hundred and thirty admissions were screened for malaria (38.7%).

Those with positive blood smears were aged 1–28 days. Forty (70.2%) of the babies were aged 7 days or less and twenty- five (43.3%) were less than 48 hours old at the time of presentation. The mean age was 6.7 days. There were 35 males and 22 females giving a male: female ratio of 1.6: 1. Infestation is not sex-linked (p-value = 0.33).

Almost forty- four percent (43.9%) of the babies weighed less than 2.5 kg and 56.1% were more than 2.5 kg at the time of presentation. Mean weight was 2.58 kg. Length of 73.2 % of the babies was less than 48 cm. Mean length was 44.29 (SD 4.33).)

As shown in table 1, 31 (54.4%) of the babies presented within 3 days of onset of illness and 52 (91%) within 7 days. The mean duration of illness was 3.6 days.

**Table 1:** Duration of Illness (days) in the babies

**Duration of Illness (Days)**	**Number of cases**	**Cumulative Total**	**Percentage%**
1–3	31		54.40
4–7	21	52	91.23
Over 7	05	57	100.0

As shown in table 2, fever was the presenting feature in 78.3 % of the babies, 6.7 % of them were hypothermic and were not feeding well. In all, 10% of them were either not tolerating feeds or refusing feeds.

**Table 2:** Frequency of Clinical Features

Clinical Features	Frequency%
Temperature> 37.2°C alone	78.3
Temperature<36.5°C+ Not feeding well	6.7
Irritability	5.0
Temperature<35.5°C alone	3.3
Diminished activity+ not feeding well	3.3
Fever, vomiting	1.7
Jitteriness	1.7

Total	100.0

Twenty- eight of the babies were delivered in public hospitals while 21 were delivered in private hospitals and the others were delivered elsewhere. The place of delivery did not significantly affect infestation. (p-value = 0.99)

While 21 of the babies were pre-term, 25 were full-term and maturity was not documented in eleven (11) of the babies. Forty- two (42) of the babies were however appropriate for age and 10 were small for date. There was no significant difference in the rate of infestation between pre-term and full-term babies (p value 0.896).

Three of the mothers were aged between 15–20 years, Seven, between 21 and 25 years and 14 were between 26–35 years. Maternal age was not documented in 23 cases. Maternal educational level was documented in only six cases. Febrile illness within the last trimester of the pregnancy was present in 30 out of the 40 cases in whom there are records. No information was however recorded for 17 cases. Infestation is significantly higher in babies of mothers with history of febrile illness (p value = 0.02).

Only 27 of the mothers were primipara, 20 were multipara, 3 Grand multipara. The parity of 7 of the mothers was not stated.

No hematological evidence of bacterial infection was found in 96.4% of the babies and thirty – seven (64.9%) had Packed Cell Volume (PCV) between 30–45%. None of them had Packed Cell Volume (PCV) less than 20%.

Table 3 shows the treatment and outcome of illness.

**Table 3:** Treatment and Outcome of Illness

Number of Patients				
Treatment	Discharged	Died	DAMA*	Total
Chloroquin 25 mg base/kg	38	2	10	50
Sulphadoxine- Pyrimethamine (SP)	1	0	0	1
No Record of Treatment	3	2	1	6

Total	42	4	11	57

*DAMA- Discharged Against Medical Advice

Fifty of the babies were treated with appropriate doses of chloroquine (25 mg chloroquine base/kg), one had Sulfadoxine-Pyrimethamine (SP). No treatment was documented in 6 cases. Forty-two of the babies responded to the antimalarial given and were subsequently discharged home in satisfactory condition. Four died and 11 were discharged against medical advice.

The duration of fever in those that died was longer than 5 days. This significantly affected the outcome of illness (p value = 0.02). Also, concurrent bacterial infection and maternal age significantly affected the outcome of illness (p values 0.02 and 0.04 respectively). Three out of the 4 deaths occurred in the babies of mothers aged 15–20 years. Apart from history of febrile illness in the mother within two weeks prior to delivery, none of the other factors examined is significantly associated with infestation.

## Discussion

This study has identified a prevalence of 24.8 % for diagnosed neonatal malaria and 17.4% for congenital malaria. This is in keeping with an African survey on congenital malaria that had reported values of 0–23% [3]. It is also noteworthy that almost half of the neonates were less than 48 hours old as at the time of presentation. Could this have been a reflection of the social class of the mothers? Unfortunately, documentation of the educational levels of the mothers was too scanty to make any reasonable deduction. However, other studies from our centre have shown that majority of mothers that attend the facility are in the low to middle socio-economic group (8). This is not unexpected considering the fact that the centre is in a semi-urban cosmopolitan setting.

Similar to our findings in this study, a report from western Uganda has documented that there is no association between congenital parasitemia and birth weight [9]. Although, over half of the babies in this review presented within three days of illness, the outcome was found to be significantly associated with the duration of illness; the longer the duration the worse the prognosis. This fact has been demonstrated even in malaria in infancy, where death from severe complicated malaria is discovered to occur within 2–3 days of illness [10]. The finding of fever as the main clinical presentation for malaria in this study is in keeping with the WHO case definition for malaria in endemic areas [11]. Interestingly, all the features found in this study are similar to those in neonatal septicaemia. A study had earlier identified that signs and symptoms of malaria in the newborn may be indistinguishable from other neonatal infections [5]. Based on this, it may be impossible to differentiate one from the other at the bedside. Considering the fatality that can result from a delay in appropriate treatment, it will appear safer to treat all cases of fever in all children, neonates inclusive, in malaria -endemic areas with antimalarials while awaiting the result of investigations.

However, that history of febrile illness in the last trimester of the pregnancy is significantly associated with infestation seem to suggest that all babies of such mothers with such history should be placed under observation in the neonatal ward and screened for malaria parasite.

The parasitology report of the six babies whose record of treatment could not be traced was probably retrieved after their exit from the hospital. They were part of the eleven that discharged against medical advice. Interestingly, they gave dissatisfaction with clinical progress of their babies as reasons for discharge. They had been on admission for some-time and the blood smear was only requested for when it appeared that the infants' condition was not improving.

Maternal age, concurrent bacterial infection and duration of illness significantly affected the outcome of illness. The three are interrelated. Babies of teenage mothers are at risk of neonatal infection. Such mothers may also present late in the hospital either due to poverty or ignorance.

In conclusion, this study has further confirmed that neonatal malaria is not as rare as we had thought in Sub-Saharan Africa. Clinicians need to have a high index of suspicion so that rapid diagnosis and early appropriate intervention can be instituted in line with the concept of Roll Back Malaria.

## Conclusion

1. All neonates with features suggestive of neonatal infection should have blood smear for malaria parasite as part of their routine screening.

2. All babies of mothers with history of febrile illness within the last two weeks of pregnancy should have blood smear for malaria parasite examination.

3. Early care seeking for neonates should be part of the educational talks in the antenatal clinics.

## Competing interests

The author(s) declare that they have no competing interests.

## Authors' contributions

TIR had the concept and wrote the article, OO analysed the data and MF entered the data. The three authors read through and agreed on the content of the article.

## Pre-publication history

The pre-publication history for this paper can be accessed here:


